# Identification and validation of coding and non-coding RNAs involved in high-temperature-mediated seed dormancy in common wheat

**DOI:** 10.3389/fpls.2023.1107277

**Published:** 2023-02-01

**Authors:** Hao Jiang, Wei Gao, Bing-li Jiang, Xue Liu, Ya-ting Jiang, Li-tian Zhang, Yue Zhang, Sheng-nan Yan, Jia-Jia Cao, Jie Lu, Chuan-xi Ma, Cheng Chang, Hai-ping Zhang

**Affiliations:** Key Laboratory of Wheat Biology and Genetic Improvement on Southern Yellow & Huai River Valley, College of Agronomy, Anhui Agricultural University, Ministry of Agriculture and Rural Affairs, Hefei, Anhui, China

**Keywords:** wheat, seed dormancy, pre-harvest sprouting, high-temperature, transcriptome sequencing

## Abstract

**Introduction:**

Seed dormancy (SD) significantly decreases under high temperature (HT) environment during seed maturation, resulting in pre-harvest sprouting (PHS) damage under prolonged rainfall and wet weather during wheat harvest. However, the molecular mechanism underlying HT-mediated SD remains elusive

Seed dormancy (SD) significantly decreases under high temperature (HT) environment during seed maturation, resulting in pre-harvest sprouting (PHS) damage under prolonged rainfall and wet weather during wheat harvest. However, the molecular mechanism underlying HT-mediated SD remains elusive.

**Methods:**

Here, the wheat landrace ‘Waitoubai’ with strong SD and PHS resistance was treated with HT from 21 to 35 days post anthesis (DPA). Then, the seeds under HT and normal temperature (NT) environments were collected at 21 DPA, 28 DPA, and 35 DPA and subjected to whole-transcriptome sequencing.

**Results:**

The phenotypic data showed that the seed germination percentage significantly increased, whereas SD decreased after HT treatment compared with NT, consistent with the results of previous studies. In total, 5128 mRNAs, 136 microRNAs (miRNAs), 273 long non-coding RNAs (lncRNAs), and 21 circularRNAs were found to be responsive to HT, and some of them were further verified through qRT-PCR. In particular, the known gibberellin (GA) biosynthesis gene *TaGA20ox1* (*TraesCS3D02G393900*) was proved to be involved in HT-mediated dormancy by using the EMS-mutagenized wheat cultivar Jimai 22. Similarly, a novel gene *TaCDPK21* (*TraesCS7A02G267000*) involved in the calcium signaling pathway was validated to be associated with HT-mediated dormancy by using the EMS mutant. Moreover, *TaCDPK21* overexpression in *Arabidopsis* and functional complementarity tests supported the negative role of *TaCDPK21* in SD. We also constructed a co-expression regulatory network based on differentially expressed mRNAs, miRNAs, and lncRNAs and found that a novel miR27319 was located at a key node of this regulatory network. Subsequently, using *Arabidopsis* and rice lines overexpressing miR27319 precursor or lacking miR27319 expression, we validated the positive role of miR27319 in SD and further preliminarily dissected the molecular mechanism of miR27319 underlying SD regulation through phytohormone abscisic acid and GA biosynthesis, catabolism, and signaling pathways.

**Discussion:**

These findings not only broaden our understanding of the complex regulatory network of HT-mediated dormancy but also provide new gene resources for improving wheat PHS resistance to minimize PHS damage by using the molecular pyramiding approach.

## 1 Introduction

Safeguarding the production of wheat (*Triticum aestivum* L.), which is the staple food for more than one-third of the human population, is directly linked to food security. Pre-harvest sprouting (PHS) means germination of physiologically mature grains in wheat spikes before harvest (Bewley, 1997). PHS negatively affects grain yield and quality as the activities of amylases, lipases, and proteases that can degrade starch, lipids, and proteins, respectively, in sprouting grains are increased during PHS. This causes global annual losses of $1 billion dollars (Vetch et al., 2019). PHS occurs frequently in regions with prolonged rainfall and wet weather during the harvest season, particularly in Canada, Australia, USA, Japan, and China (Liu et al., 2008; Rajjou et al., 2012; ). In China, PHS affects a total of 24.91 million hectares, which is 83% of the wheat planting area, especially in the northern spring wheat region, Yangtze River Valley, and northeastern spring wheat region. These regions are characterized by frequent rainfall and high humidity during harvest (Xiao et al., 2002). PHS has also recently become a serious problem in the Yellow and Huai Valleys’ wheat region because of climate changes (Ali et al., 2019). Therefore, breeding wheat varieties with high PHS resistance is the major target in regions that receive frequent rainfall and have wet weather before harvest.

Seed dormancy (SD) is defined as temporarily blocking of germination of viable and healthy seeds under favorable conditions. SD is considered a major genetic factor for improving PHS resistance, and the occurrence of SD and its intensity are partially modified by environmental factors (such as temperature, light, and oxygen) (Naylor and Jana, 1983). Temperature is regarded the most influential factor among SD-controlling external conditions. Reddy et al. (1985) found that wheat cultivars (P.I. 178211, Brevor, and Tom Thumb) with strong dormancy developed at 26°C were more likely to germinate in seeds than at 15°C. Rodríguez et al. (2001) showed that barley plants grown at >20°C contributed to break SD and promote seed germination. Biddulph et al. (2007) demonstrated that SD in wheat reduced significantly when the seeds were subjected to sudden heat shocks (>30°C max. for >12 days) at 30–50 days post anthesis (DPA), and the higher the number of days with the maximum temperature of >30°C, the lower the dormancy level. Jiménez et al. (2017) reported a positive correlation between temperature and susceptibility to PHS. Briefly, these results demonstrated that high temperature (HT) during seed maturation obviously reduces the dormancy level. However, the detailed molecular mechanism underpinning HT-mediated dormancy remains unclear. Notably, extreme heat conditions may occur frequently with global warming, posing a potential risk to wheat production when rainfall and wet weather occur during harvest (Telfer et al., 2018). Therefore, comprehensively and systematically dissecting the molecular network of HT-mediated dormancy during seed maturation is necessary, and this will contribute to the development of high PHS-resistant varieties for coping with prolonged rainfall and wet weather.

Until now, seven genes controlling SD and PHS resistance have been identified through map- or homology-based cloning in wheat, namely *TaVp-1* (Yang et al., 2007), *TaDOG1L1* (Ashikawa et al., 2014), *TaMFT/TaPHS1* (Nakamura et al., 2011; Liu et al., 2013), *TaSdr* (Zhang et al., 2014; Zhang et al., 2017), *TaMKK3* (Torada et al., 2016), *TaQsd1* (Wei et al., 2019) and *Myb10-D* (Lang et al., 2021). Among them, *TaDOG1L1* is a wheat homolog of the *Arabidopsis* dormancy gene *DOG1* (*Delay of Germination 1*). *DOG1* is the first gene associated with natural variation in primary dormancy in *Arabidopsis* (Bentsink et al., 2006). *DOG1* expression increased when seeds matured at low temperature, and this increased expression led to increased dormancy of *Arabidopsis* seeds (Chiang et al., 2011). Further, *DOG1* expression led to the increase in SD at low temperature by promoting phytohormone gibberellin (GA) catabolism (Kendall et al., 2011). Similarly, the expression of *MFT-3A* (*TaMFT/TaPHS1*), a wheat homolog of *MOTHER OF FT AND TFL1* (*MFT*), was upregulated after physiological maturity in dormant seeds grown at a lower temperature, and may suppress GA synthesis in the scutellar epithelium and finally induce dormancy in wheat (Nakamura et al., 2011). Thus, these results indicate that *TaDOG1L1* and *TaMFT/TaPHS1* are involved in temperature-regulated SD. However, whether additional genetic factors, including coding and non-coding RNAs (ncRNAs), participate in the complex network of HT-mediated dormancy during seed maturation in common wheat remains unclear.

NcRNAs are transcripts that are not translated into proteins and play key roles in various plant biological processes. They mainly include microRNAs (miRNAs), long non-coding RNAs (lncRNAs), and circular RNAs (circRNAs) (Martin et al., 2010; Ma et al., 2015). Until now, many miRNAs have been identified to be associated with SD and seed germination in different plant species, including miR160 (Liu et al., 2007), miR159 (Reyes and Chua, 2007), miR395c and miR395e (Kim et al., 2010) in *Arabidopsis*; miR156 and miR172 (Huo et al., 2016) as well as miR408 (Jiang et al., 2021) in *Arabidopsis* and lettuce, respectively; miR9678 in wheat (Guo et al., 2018); and miR156 in rice (Miao et al., 2019). Notably, Huo et al. (2016) found that the known dormancy gene *DOG1* regulated SD by influencing miR156 and miR172 levels in *Arabidopsis* and lettuce, respectively. In addition, a few lncRNAs were reported to be involved in SD and seed germination. Guo et al. (2018) reported that miR9678 targeted a lncRNA (WSGAR) and triggered phased small interfering RNA (siRNA) generation, eventually mediating SD and seed germination in wheat. Wu et al. (2018) reported that cabbage BoNR8 lncRNA was associated with SD in transgenic *Arabidopsis* plants. However, the association of circRNAs with SD and seed germination has not yet been reported. Taken together, these findings demonstrate that miRNAs and lncRNAs are crucial players in SD and seed germination. However, whether ncRNAs, as crucial regulators, participate in HT-mediated dormancy in common wheat needs to be clarified.

The objectives of this study were as follows: (i) investigate changes in dormancy of the wheat landrace Waitoubai (WTB) exhibiting high dormancy and PHS resistance (Xiao et al., 2002) under HT and normal temperature (NT) conditions during seed maturation; (ii) determine differences in the expression of global mRNAs, miRNAs, lncRNAs, and circRNAs in WTB seeds after NT and HT treatments through whole-transcriptome sequencing and validate expression patterns of some representative mRNAs, lncRNAs, miRNAs, and circRNAs through qRT-PCR; (iii) preliminarily validate the roles of representative mRNAs and miRNAs in SD in transgenic *Arabidopsis* and rice lines as well as ethyl methane sulphonate (EMS)-mutagenized wheat cultivar Jimai 22 (JM22); and (vi) analyze the expression of key genes involved in the abscisic acid (ABA) and GA biosynthesis/catabolism/signaling pathways and ABA/GA contents in the transgenic and wild-type rice seeds. These results can provide new insights into the complex molecular network of HT-mediated SD in wheat.

## 2 Materials and methods

### 2.1 Sample preparation and growth conditions

The wheat landrace WTB with high dormancy and PHS resistance was subjected to HT and NT treatments, as well as whole-transcriptome sequencing. The WTB plants were potted in a natural field environment (31°58′N, 117°240′E, Hefei, China, **Supplemental Table 1**) during 2017–2018 cropping seasons before anthesis. All fields were maintained as disease- and weed-free. After selecting the representative 60 main stem spikes to mark in anthesis, the plants were transferred to two illumination incubators for HT (35°C day/25°C night) or NT (25°C day/20°C night) treatment (16 h day/8 h night, 70% relative humidity) from 21 DPA to 35 DPA. Seeds were collected at 21 DPA, 28 DPA, and 35 DPA, named 21DPA-NT, 28DPA-NT, 28DPA-HT, 35DPA-NT, and 35DPA-HT; immediately frozen in liquid nitrogen; and stored at −80°C for RNA extraction.

The *TaGA20ox1* mutant (designated *ga20ox1*, jm_chr3D_508613303) and the *TaCDPK21* mutant (designated *cdpk21*, jm_chr7A_270043204) of wheat cultivar JM22, created using EMS provided by Yantai Jien Biotechnology Company (http://jm.ytjebc.com/), were used to determine the seed germination percentage (GP). The *cdpk21* mutant plants were grown in growth chambers (16 h day/8 h night, 70% relative humidity). The *ga20ox1* mutant plants were also grown in growth chambers (16 h day/8 h night, 70% relative humidity) and treated at HT (NT as control) from 21 DPA to 35 DPA, which is consistent with the treatment methods used for the WTB plants.

All *Arabidopsis* plants used had the *Arabidopsis thaliana* Columbia (Col-0) accession background. The *atcdpk24* mutant (SALK_015986C) was ordered from The Arabidopsis Information Resource (TAIR, https://www.Arabidopsis.org/). Homozygous mutant plants were identified in the F_2_ generation through PCR by using primers obtained from the Salk Institute Genomic Analysis Laboratory (SIGnAL) database (**Supplemental Table 2**). For planting, seeds were imbibed at 4°C for 3 days for stratification and were harvested from plants cultivated in soil in a greenhouse at 21°C ± 1°C (16 h day/8 h night, 60% relative humidity).

Rice (*Oryza sativa* L. *japonica* Nipponbare) plants were grown in a walk-in growth chamber under a 12-h light (28°C)/12-h dark (22°C) photoperiod regime. Homozygous lines (T_2_ generation) were selected for the subsequent experiments.

### 2.2 Seed dormancy assay

GP was used to evaluate SD. Wheat seed GP was determined according to the method of Zuo et al. (2019). Germinated seeds were selected on the basis of seed coat rupture at the embryo site, and GP was calculated as the number of germinated seeds by the 7th day of the germination test divided by the total number of seeds.

For *Arabidopsis* seeds, germination phenotypes were determined using the method of Cao et al. (2020). Briefly, approximately 50 seeds of individual plants were sown on filter paper saturated with sterile distilled water in Petri dishes and cultivated in a growth chamber (16 h light/8 h dark, 21°C ± 1°C). The seeds were collected at 25 DPA, and the GP was measured on the 7th day of the germination assay. Seeds with a protruded radicle were considered to be germinated.

For rice seeds, germination phenotypes were determined using the method of Li et al. (2020). Rice seeds with a ≥ 1-mm-long radicle were considered to be germinated.

### 2.3 Plant hormones and α-amylase activity measurements

To determine ABA, GA, and α-amylase activities, freshly harvested rice seeds were imbibed in the growth chamber (16 h light/8 h dark, 21°C ± 1°C) for 24 h before sampling. Approximately 100 mg samples of the imbibed seeds were used. ABA and GA levels were quantified using the ELISA Kit (Meimian, BY-JZF0146 and BY-JZF0147) according to the manufacturer’s instructions. Alpha-amylase activity was measured using a kit (Solarbio, BC0615) according to the manufacturer’s instructions.

### 2.4 RNA extraction and sequencing library construction

Total RNA was isolated from 15 samples (21DPA-NT, 28DPA-NT, 28DPA-HT, 35DPA-NT, and 35DPA-HT, 3 repeats for each sample) by using the MiniBEST Plant RNA Extraction Kit (TaKaRa) in the same manner as for transcriptome sequencing. The complementary DNA (cDNA) library was constructed using the Ribo-Zero Magnetic Kit (Madison, USA). The procedure was performed in accordance with that described in a study by Zhang et al. (2016). Libraries for small RNA sequencing were constructed using the NEB NextR UltraTM small RNA sample library prep kit (NEB, USA). Strand-specific sequencing for mRNAs and small RNAs was performed on an Illumina HiSeq4000 system by Biomarker technologies (Beijing, China); sequencing was performed using the standard Illumina protocol according to Ma et al. (2015).

### 2.5 Transcriptome analysis

Reference genome sequences and annotations were downloaded from the wheat IWGSC v1.1 genome website (https://urgi.versailles.inra.fr/download/iwgsc/IWGSC_RefSeq_Annotations/v1.1/). Reads containing the adaptor sequence, duplicate sequence, bases with a quality score of < Q10 more than 50%, and with uncertain bases (reads with > 10% unknown sequences “N”) were removed. For the RNA-seq data, the clean reads were filtered from the raw reads mapped to the wheat reference genome by using Bowtie2 (http://bowtie-bio.sourceforge.net/bowtie2) (Langmead and Salzberg, 2012) and HISAT2 (http://www.ccb.jhu.edu/software/hisat) (Kim et al., 2015) with default parameters. The Fragments Per Kilobase of transcript per Million fragments mapped (FPKM) method was used to measure transcript expression levels by using Cufflinks software (http://cole-trapnell-lab.github.io/cufflinks) (Trapnell et al., 2012). Reads uniquely mapped to the reference sequences (with ≤ 1 mismatch) and FPKM ≥ 1 in at least one sample were used for identifying differentially expressed genes (DEGs). DEG analysis was performed using DEGseq (https://bioconductor.org/biocLite.R) with criteria of fold change ≥ 2 and *P* < 0.01 (Wang et al., 2010). Kyoto Encyclopedia of Genes and Genomes (KEGG) analyses were performed to functionally categorize DEGs by using the KEGG (http://www.genome.jp/kegg/genes.html) databases, respectively (Kanehisa and Goto 2020). TBtools software (https://github.com/CJ-Chen/TBtools) (Chen CJ et al., 2020) was used to identify the overlapping differentially expressed RNAs in different samples and tissues. Transcription factor (TF)-related sequence information was obtained from the Plant Transcription Factor Database (http://planttfdb.gao-lab.org) (Jin et al., 2015).

### 2.6 LncRNA analysis

Following the assembly, cuffcompare (a tool of Cufflinks, http://cole-trapnell-lab.github.io/cufflinks) was used to investigate the location between transcripts and known mRNAs and lncRNAs (Trapnell et al., 2012). The non-coding transcripts were identified using four software, including Coding Potential Calculator2 (http://cpc2.gao-lab.org/) (Kang et al., 2017), Coding-Non-Coding Index (https://github.com/www-bioinfo-org/CNCI) based on the coding potentials that scored < 0 (Sun et al., 2013), Coding Potential Assessment Tool (CPAT, http://lilab.research.bcm.edu) (Wang et al., 2013) based on the length, and coverage of the Pfam protein database (http://pfam.xfam.org/) (Mistry et al., 2020) based on no transcripts was aligned. The lncRNAs were identified when they satisfied all methods and were classified into several categories according to their genomic location and previous description of the Rfam by Inference of RNA Alignments (INFERNAL, http://eddylab.org/infernal/) (Nawrocki and Eddy, 2013). Coding genes located within 100-Kb upstream and downstream of lncRNAs were identified as *cis*-targets.

### 2.7 CircRNA analysis

To obtain a set of predicted circRNA loci from the RNA-seq data, Find_circ v1.2 (https://github.com/marvin-jens/find_circ) (Memczak et al., 2013) with output filtered for a minimum of 6 reads was used. The raw counts were first normalized using transcripts per million (TPM). CircRNAs with fold changes ≥ 2 and *P* ≤ 0.05 were classified as circRNAs with significant differential expression.

### 2.8 MicroRNA analysis

Following the trimming of adaptor sequences in the 5’ and 3’ ends of sequenced reads, we filtered out low-quality reads (reads with unknown sequences “N”), adaptor sequence fragments, and empty reads. By using the SOAP2 (http://soap.genomics.org.cn) (Li et al., 2009) with parameter settings for perfect matches, reads were aligned to the genome. Then, the unique sequences of length 18–25 nucleotides (nt) were blasted to rice precursors in miRBase v22 (https://www.mirbase.org/) (Kozomara et al., 2019) to detect known miRNAs. Mireap v0.1 (https://help.rc.ufl.edu/doc/Mireap) (Li et al., 2011) was used for predicting novel miRNAs. TPM was used to normalize the counts, and miRNAs with fold changes ≥ 2 and *P* ≤ 0.05 were categorized as miRNAs with significant differential expression. Target genes of differentially expressed miRNAs were predicted using psRNA Target (http://plantgrn.noble.org/psRNATarget) (Dai and Zhao, 2011).

### 2.9 qRT-PCR assay

A set of selected DEGs from differentially expressed transcripts were validated through qRT-PCR by using the same RNA samples as those used for transcriptome sequencing. For transgenic materials, seeds imbibed for 24 h were used for quantitative analysis. The target gene sequences were used to design gene-specific primer pairs by using Primer Premier v5.0 (**Supplemental Table 2**). The SYBR green Premix Ex TaqTM II (TaKaRa, RR081A) quantitative PCR system was used for qRT-PCR analysis. Approximately 0.5 μg of isolated RNA was used for first-strand cDNA synthesis by using the Prime Script RT reagent kit (Takara, RR037A) following the manufacturer’s instructions for mRNAs and lncRNAs. For cirRNAs, RNase R (Jisai, R0301) was used to remove genomic linear DNA before reverse transcription. Furthermore, instead of oligo (dT) primers, random primers were added to synthesize first-strand cDNA for circRNAs. The method of poly (A) tail was used for miRNA quantitative confirmation, and the miRNA First-Strand Synthesis Kit (Takara, No.638315 and No.638316) was used based on the manufacturer’s instructions. The following amplification program was used: denaturation at 95°C for 2 min and 40 cycles of amplification (95°C for 10 s, followed by 59°C for 30 s). Melting curve analysis was performed from 65°C to 95°C, with 5 s increments of 0.5°C. The wheat *Actin* gene was used as an internal control for mRNAs, lncRNAs, and circRNAs. The wheat *U6* gene was used as an internal control for miRNAs. All samples were analyzed in triplicate. The 2^-△△Ct^ (Livak and Schmittgen, 2001) relative quantification method was used to calculate the relative expression level.

### 2.10 Plasmid construction and transformation

The *TaCDPK21-7A* (*TraesCS7A02G267000*) cDNA sequence from ‘Chinese Spring’ was amplified and cloned into the *Eco*R I and *Bam*H I sites of the binary vector pCAMBIA1301a under the control of the 35S promoter. The vector was transformed into the *Agrobacterium tumefaciens* strain CV3101. Subsequently, it was transformed into *Arabidopsis* Col-0 and *atcdpk24* plants by using previously described methods (Clough and Bent, 1998).

The miR27319 full-length sequence (approximately 300 nt before and after the miRNA site) from ‘Chinese Spring’ was amplified and cloned into the *Eco*31 I sites of the binary vector pCAMBIA1300 under the control of the 35S promoter. To create a vector of short tandem target mimic (STTM), a 48-nt-specific sequence of miR27319 (with both ends including miRNA target binding sites and *Eco*31 I sites) was recombined into the expression vector pCAMBIA1300. These vectors were transformed into the *A. tumefaciens* strain CV3101 and subsequently transformed into mature rice seed-derived calli (Nipponbare, Nip) (Hiei et al., 1994) and into the *Arabidopsis* Col-0 plants (Clough and Bent, 1998) by using previously described methods. The transgenic plants were selected using hygromycin, and transgene insertion was validated through DNA extraction and PCR (**Supplemental Table 2**).

### 2.11 Accession numbers and statistical analysis

Clean Illumina sequences were deposited in the National Center for Biotechnology Information Databank (NCBI) (accession number: PRJNA895954).

SPSS v22.0 (http://www.spss.com) software was used for analyzing phenotypic data, and correlation coefficients were estimated using the Pearson method. The Mann–Whitney U-test was performed through stepwise, step-down comparisons.

## 3 Results

### 3.1 High temperature repressed seed dormancy in ‘Waitoubai’

The GP values of the seeds collected from 28DPA-NT, 35DPA-NT, 28DPA-HT, and 35DPA-HT were 0%, 0%, 9%, and 63%, respectively (**Supplemental Table 3**), indicating that HT obviously reduces the SD level and promotes germination compared with NT, which is consistent with the previous study results.

### 3.2 Assessing the overall quality of sequencing data

As shown in **Supplemental Table 4**, 2070.31 million reads in chain-specific libraries were obtained. Of them, 2063.48 million reads were clean reads, accounting for 99.67% of the total reads. Average reads of 85.18% were aligned to the wheat reference genome (**Supplemental Table 4**). Pearson correlation analysis showed a good relationship for biological replicates (0.952-0.993**, **Supplemental Table 5**). In total, 130312 mRNAs were obtained, 41316 of which were identified as new genes (**Supplemental Table 6**). Additionally, we obtained 40970 lncRNAs and 4414 circRNAs (**Supplemental Tables 6**, **7**). Overall, most lncRNAs were approximately 300-500 nt long (**Figure S1A**), and the expression levels of lncRNAs were generally lower than those of mRNAs (**Figure S1B**). The percentages of lncRNA classes suggested that most lncRNAs were long intergenic-lncRNAs (lincRNAs) (86.5%), followed by antisense-lncRNAs (8.2%), intronic-lncRNAs (2.7%), and sense-lncRNAs (2.6%) (**Figure S1C**). CircRNAs were mainly produced from genes and most of them were approximately 200-400 nt long (**Figure S2A**). In total, 2439 circRNAs were derived from the gene ID in IWGSC v1.1, and 1166 circRNAs were derived from new genes predicted in this study (**Figure S2B**).

In total, 344.62 million reads were obtained from miRNA libraries, and 191.41 million clean reads were obtained (**Supplemental Table 4**). Because primary miRNA transcripts contain one or more miRNA stem loops that could be processed into mature miRNAs, 785 miRNAs were identified from these libraries, including 79 known and 706 novel miRNAs (**Supplemental Table 6**). The majority of the small RNAs were 21- and 24 nt long (**Figure S2C**). The proportions of first base distribution are shown in **Figure S2D**; the proportion of the U base (47%) was higher than those of A (33%), C (14%), and G (6%).

### 3.3 Differentially expressed RNAs of HT-mediated SD

**Figure 1:**
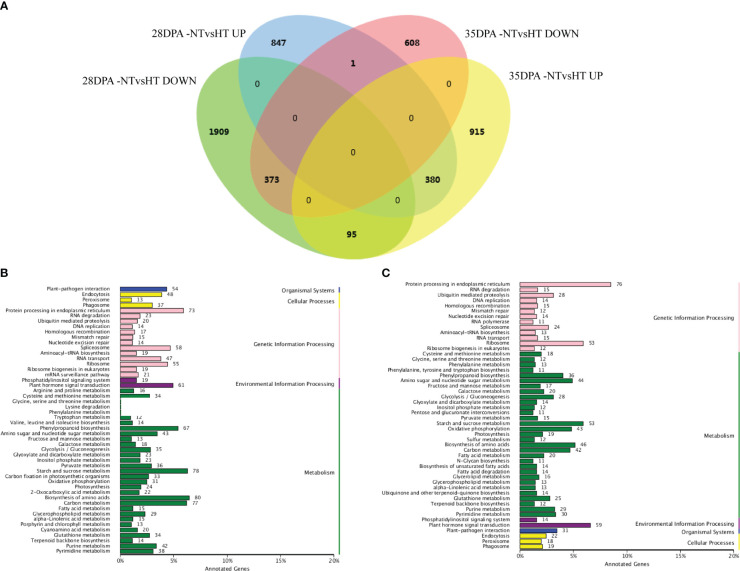
Differentially expressed mRNAs and KEGG analysis. **(A)** Venn diagram of differentially expressed mRNAs. **(B)** The KEGG analysis of differentially expressed mRNAs identified in 28DPA-NTvsHT. **(C)** The KEGG analysis of differentially expressed mRNAs identified in 35DPA-NTvsHT.

Overall, 3605 and 2372 mRNAs were differentially expressed in 28DPA-NTvsHT and 35DPA-NTvsHT, respectively (**Figure 1A**; **Supplemental Table 8**). Of them, 373 down-regulated mRNAs and 380 up-regulated mRNAs were common to the two groups (**Figure 1A**). In 28DPA-NTvsHT, DEGs were involved in benzoxazinoid biosynthesis; valine, leucine, and isoleucine biosynthesis; starch and sucrose metabolism; and carbon fixation in photosynthetic organisms, as determined through KEGG enrichment analysis (**Figure 1B**). In 35DPA-NTvsHT, DEGs were involved in protein processing in the endoplasmic reticulum and in amino sugar and nucleotide sugar metabolism (**Figure 1C**).

According to the Venn diagram, 94 and 66 miRNAs were differentially expressed in 28DPA-NTvsHT and 35DPA-NTvsHT, respectively. Of them, 14 down-regulated miRNAs and 10 up-regulated miRNAs were common to the two groups (**Figure S3A**; **Supplemental Table 9**). In total, 174 and 137 lncRNAs were differentially expressed in 28DPA-NTvsHT and 35DPA-NTvsHT, respectively. Of them, 28 down-regulated lncRNAs and 3 up-regulated lncRNAs were common to the two groups (**Figure S3B**; **Supplemental Table 10**). In addition, 13 and 11 circRNAs were differentially expressed in 28DPA-NTvsHT and 35DPA-NTvsHT, respectively. Only 3 up-regulated circRNAs were common to the two groups (**Figure S3C**; **Supplemental Table 11**).

### 3.4 Constructing the regulatory network of HT-mediated SD

To determine the functional relationships between ncRNAs and the target genes involved in HT-regulated dormancy, we constructed a regulatory network based on differentially expressed mRNAs, miRNAs, and lncRNAs (**Figure S4**; **Supplemental Table 12**). The network map revealed that the relationships between coding and non-coding RNAs were one-to-one, one-to-many, or many-to-one; otherwise, lncRNAs could act as a common target for miRNAs and mRNAs. Among the 849 differentially expressed mRNAs identified in both 28DPA-NTvsHT and 35DPA-NTvsHT, 374 contained miRNA identical regions, which implied that these mRNAs may contain miRNA-binding sites and act as targets for miRNAs (**Supplemental Table 8**). In the regulatory network, unconservative_chr5A_27319 (designated miR27319) was particularly located at the key node, suggesting its key role in HT-mediated dormancy; however, this result needs to be further validated experimentally (**Figure S4**; **Supplemental Table 12**). Moreover, 6 lncRNAs were found to be co-expressed with 7 mRNAs, including 5 involved in cis-regulatory mechanisms and 2 trans-regulatory mechanisms (**Supplemental Table 12**).

### 3.5 qRT-PCR validation

To validate transcriptome sequencing results, qRT-PCR was performed to determine the expression patterns of randomly selected 64 mRNAs, 11 miRNAs, 50 lncRNAs, and 5 circRNAs from the 35DPA-NT and 35DPA-HT samples. As shown in **Figures S5A, B**, and **Supplemental Table 13**, the expression of all these selected mRNAs exhibited the same change trends determined through RNA-seq analysis, indicating that the transcriptome sequencing results were reliable. The aforementioned selected candidates were involved in various biological pathways, including 25 genes encoding TFs (such as C3H, MYB, bHLH, MADS, WRKY, GATA, and B-box), 17 genes involved in plant hormone pathways (such as ABA, GA, ethylene, and auxin pathways), 4 peroxidase genes, 2 heat shock protein family genes, 3 heat stress TF genes, 5 cytochrome P450 family genes, and 8 genes involved in other pathways (**Supplemental Table 13**).

The expression patterns of 9 of the 10 miRNAs were consistent with the sequencing data (**Figure S5C**; **Supplemental Table 13**). According to both the sequencing data and qRT-PCR results, the expression of the known tae-miR408 and tae-miR395b was downregulated in 35DPA-NTvsHT, whereas that of the three reported miRNAs (tae-miR9779, tae-miR9772, and tae-miR9774) was upregulated, indicating that these miRNAs are responsive to HT (**Figure S5C**; **Supplemental Table13**). Additionally, 4 novel miRNAs (unconservative_chr1A_1868, unconservative_chr5A_27319, unconservative_chr5A_26651, and unconservative_chr3A_15287) were validated to be differentially expressed after HT treatment (**Supplemental Table 13**).

The expression patterns of 50 lncRNAs with a high fold change in 35DPA-NTvsHT were consistent with those obtained from the sequencing data (**Figure S5D** and **Supplemental Table 13**). In particular, five lncRNAs (MSTRG.1841654.4, MSTRG.3326929.1, MSTRG.3111190.1, MSTRG.918018.1, and MSTRG.364559.2) exhibited more than 3-fold differences between 35DPA-NT and 35DPA-HT in both RNA-seq analysis and qRT-PCR, implying their critical roles in HT-mediated dormancy (**Supplemental Table 13**).

The primers for circRNAs were designed for qRT-PCR validation. Five selected circRNAs (chr2D:624323990|624324666, chr2D:624324010|624324666, chr3B:127321663|127322231, chr3D:866248|867183, and chr3D:477885083|477889020) were observed to be processed through back-splicing, confirming the reliability of high-throughput transcriptome sequencing data (**Figure S5E**; **Supplemental Table 13**).

### 3.6 Known dormancy-related genes and predicted non-coding RNAs

According to Tai et al. (2021), 81 SD-associated genes have been summarized in wheat, with eight of them being differentially expressed in WTB seeds after HT treatment. Expression of the four known dormancy genes (*TaDOG1-3A*, *TaDOG1-3D*, *TaMFT-3A*, and *TaMFT-3D*) was downregulated in the WTB seeds after HT treatment, which was further validated through qRT-PCR (**Supplemental Table 13**). The other four known genes (*TaSnRK2-2D*, *TaAFP-2D*, *GID1-1A*, and *GID1-1D*), which are involved in the ABA and GA signaling pathways, were also responsive to HT in the WTB seeds. This suggested that ABA and GA signals may participate in HT-mediated dormancy (**Supplemental Table 14**).

Notably, *TaDOG1-3A*, *GID1-1D*, *TaMFT-3A*, and *TaMFT-3D* were predicted to be targeted by the novel lncRNAs MSTRG.4103020.1, MSTRG.861889.1, MSTRG.729470.1, and MSTRG.891662.2, respectively. This will contribute to broaden our understanding of the molecular mechanisms of *TaDOG1* and *TaMFT* in SD regulation (**Supplemental Table 14**).

### 3.7 Validation of *TaGA20ox1* involved in HT-mediated SD

**Figure 2:**
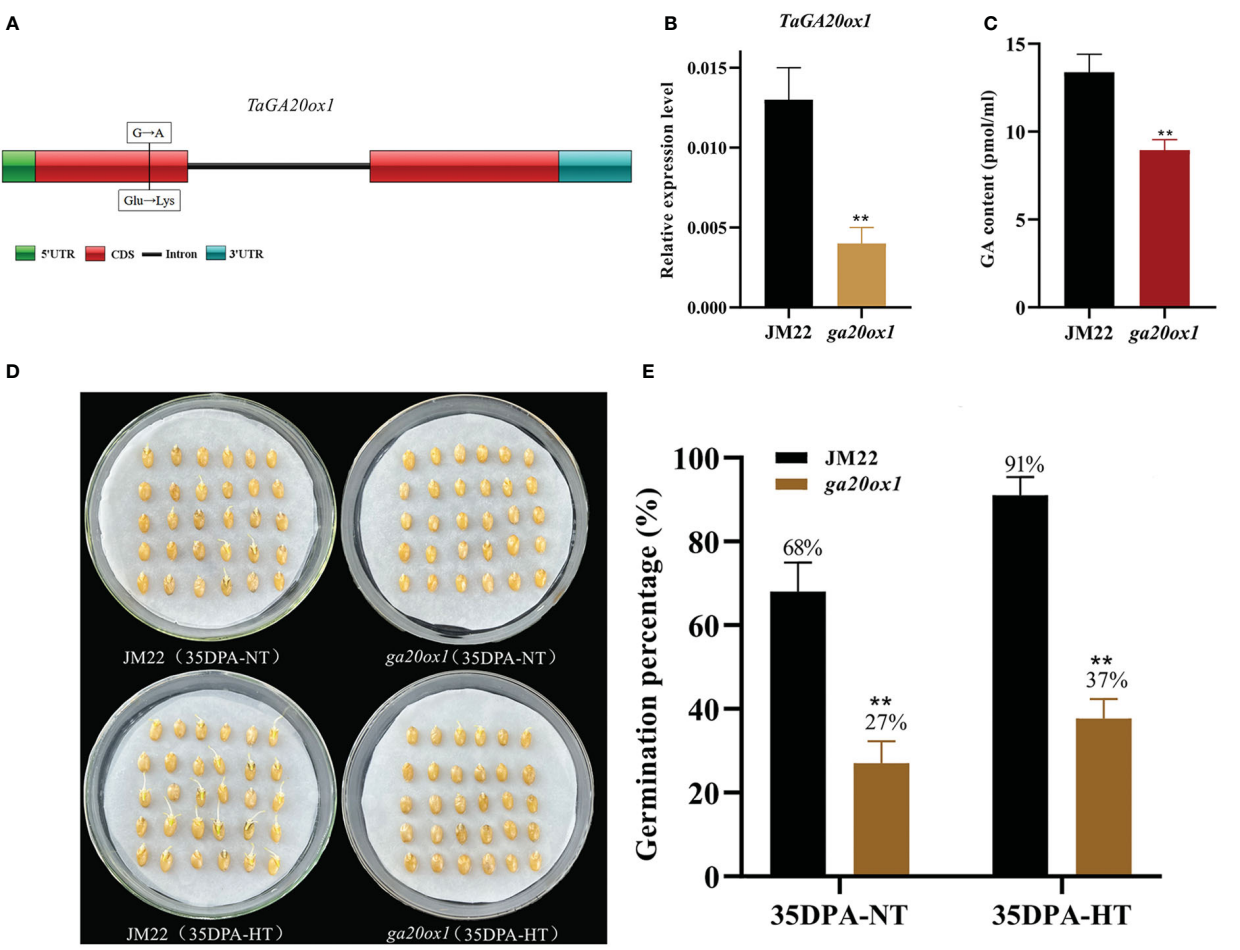
Mutation site, GA contents and germination percentages of the EMS mutant *ga20ox1.* **(A)** Mutation site of *ga20ox1*. **(B)** Relative expression levels of GA20ox1 in mutant lines and JM22. Significant differences were determined using Student’ s t-test: **P < 0.01. **(C)** GA contents of Jimai 22 (JM22) and *ga20ox1* seeds at 96 h after imbibition. **(D)** The seed images of Jimai 22 (JM22) and the EMS mutant *ga20ox1* after 96 h imbibition conducted under normal-temperature (25°C/20°C) and high-temperature (35°C/25°C) environments. **(E)** Germination percentages of JM22 and the EMS mutant *ga20ox1* on the 7th day of germination tests conducted under normal-temperature (25°C/20°C) and high-temperature (35°C/25°C) environments. Data represent the mean ± standard error (SE), n = 10–15.

HT significantly upregulated the expression of *gibberellin 20 oxidase 1-B-like* gene (designated *TaGA20ox1*, *TraesCS3D02G393900*) (4.87-fold in 28DPA-NTvsHT and 2.84-fold in 35DPA-NTvsHT), and its expression pattern was verified through qRT-PCR (**Figure S6A**). To validate the role of *TaGA20ox1* in HT-mediated SD, we obtained the EMS mutant *ga20ox1* with a SNP mutation (G/A) at the 128th amino acid in the functional domain (a non-heme dioxygenase N-terminal domain) of *TaGA20ox1*. This resulted in the conversion from glutamate (Glu) to lysine (Lys) in the wheat cultivar JM22 (**Figure 2A**). To avoid the interference of the genetic background, the *ga20ox1* mutant was backcrossed with JM22 for two generations. In the BC_2_F_2_ (*ga20ox1*) mutant seeds, the relative expression of *GA20ox1* was significantly decreased in mutant lines compared with JM22 (**Figure 2B**). And the endogenous GA content of *TaGA20ox1* was significantly decreased in *ga20ox1*, revealing that the *TaGA20ox1* mutation affects endogenous GA biosynthesis (**Figure 2C**). The *ga20ox1* seeds exhibited obviously lower GPs (average GP 27%) than JM22 seeds (average GP 68%) (**Figures 2D, E**). Moreover, compared with JM22 seeds (average GP 91%), *ga20ox1* seeds were less sensitive to HT (average GP 37%) (**Figures 2D, E**). These results confirm that *TaGA20ox1*, as a crucial component in the GA biosynthesis pathway, participates in HT-mediated dormancy.

### 3.8 Validation of *TaCDPK21* involved in HT-mediated SD

The calcium signaling pathway is known to be related to SD and seed germination (Zhu et al., 2007; Zhao et al., 2011). Being a calcium sensor, the expression of the gene *calcium-dependent protein kinase 21-like* (designated *TaCDPK21*, *TraesCS7A02G267000*) was significantly upregulated (1.69-fold) in 35DPA-NTvsHT. The *TaCDPK21* expression pattern in 35DPA-NTvsHT was further verified through qRT-PCR (**Figure S7A**). Combining increased GP and decreased SD phenotypes, we suggest that *TaCDPK21* negatively regulates SD and positively regulates germination.

**Figure 3:**
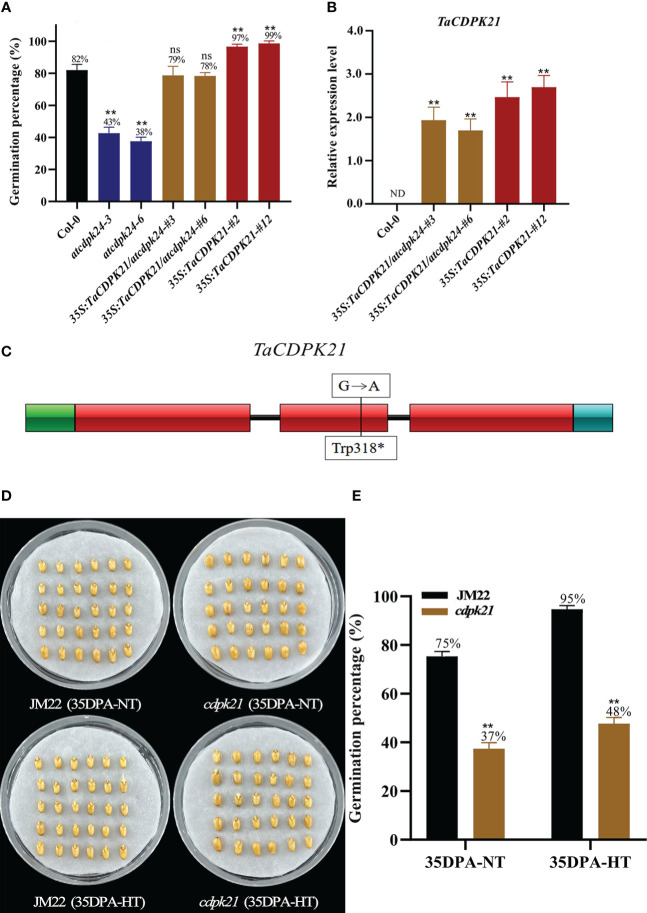
*TaCDPK21* significantly and negatively regulates seed dormancy. **(A)** Germination percentages of Col-0, the T-DNA mutant *atcdpk24*, overexpression plants (*35S:TaCDPK21*), and complementation of *TaCDPK21* (*35S:TaCDPK21/atcdpk24*) in *Arabidopsis*. Data are the mean ± standard error (SE), n = 10–15. Significant differences were determined using Student’ s t-test: **P < 0.01. ns represents not significant **(B)** Relative expression of *TaCDPK21* in Col-0, overexpression *Arabidopsis* plants (*35S:TaCDPK21*), and complementation of *TaCDPK21* (*35S:TaCDPK21/atcdpk24*). RNA was extracted from seeds imbibed for 24 h. ND represents not detected. **(C)** Mutation site of *cdpk21*. * represents terminator **(D)** The seed images of Jimai 22 (JM22) and the EMS mutant (*cdpk21*) after 96-h imbibition conducted at normal temperature (25°C/20°C). **(E)** Germination percentages of JM22 and the EMS mutant (*cdpk21*) on the 7th day of germination tests conducted at normal temperature (25°C/20°C) and high-temperature (35°C/25°C) environments. Significant differences were determined using Student’ s t-test: **P < 0.01. Data are the mean ± standard error (SE), n = 10–15.

To preliminarily investigate the role of *TaCDPK21* in SD, we searched its *Arabidopsis* homologous AT2G31500 (*AtCDPK24*) on the basis of the protein sequence blast (http://plants.ensembl.org/index.html) and obtained the mutant *atcdpk24* (SALK_015986C). This mutant had a transfer DNA (T-DNA) inserted in the third exon of *AtCDPK24* and lacking *AtCDPK24* expression (**Figure S7B**). According to germination test results, the GP (average GP 41%) of *atcdpk24* mutant lines were lower than those of Col-0 (average GP 82%) (**Figure 3A**), demonstrating that *AtCDPK24* has a negative role in SD. Considering the homologous relationship between them, we speculate that *TaCDPK21* may have a function similar to that of *AtCDPK24*. Subsequently, we constructed a fusion with the full-length coding sequence of *TaCDPK21* under the control of the constitutive cauliflower mosaic virus 35S promoter for overexpression in the *atcdpk24* mutant background (*35S:TaCDPK21/atcdpk24*). Two independent transgenic lines (*35S:TaCDPK21/atcdpk24-#3* and *35S:TaCDPK21/atcdpk24-#6*) were selected for further studies (**Figures 3B**, **S7C**). The phenotypic data revealed that *TaCDPK21* overexpression increased the GPs (average GP 79%) of the *atcdpk24* mutant lines (average GP 41%), suggesting that *TaCDPK21* negatively regulates SD and positively mediates germination (**Figure 3A**). Simultaneously, transgenic *Arabidopsis* plants ectopically expressing *TaCDPK21* were also generated (*35S:TaCDPK21-#2* and *35S:TaCDPK21-#12*) (**Figures 3B**, **S7C**). According to the results, the GP of the *35S:TaCDPK21* plants (average GP 98%) were higher than those of Col-0 (average GP 82%). This finding supports the negative role of *TaCDPK21* in SD and the positive role in germination (**Figure 3A**).

To authenticate the roles of *TaCDPK21* in wheat SD, we identified a *cdpk21* EMS mutant (JM22) with a point mutation (G/A) in the protein kinase domain site of *TaCDPK21*, resulting in premature termination of translation (**Figure 3C**). To eliminate the interference of the genetic background, the *cdpk21* mutant was backcrossed with JM22 for two generations. The BC_2_F_2_ (*cdpk21*) mutant seeds showed lower GPs (average GP 37%) than JM22 seeds (average GP 75%) (**Figures 3D, E**). Moreover, compared with JM22 seeds (average GP 95%), *cdpk21* seeds were less sensitive to HT (average GP 48%) (**Figures 3D, E**). The results confirm that *TaCDPK21* participates in HT-mediated dormancy.

### 3.9 Validation of miR27319 related to SD

**Figure 4:**
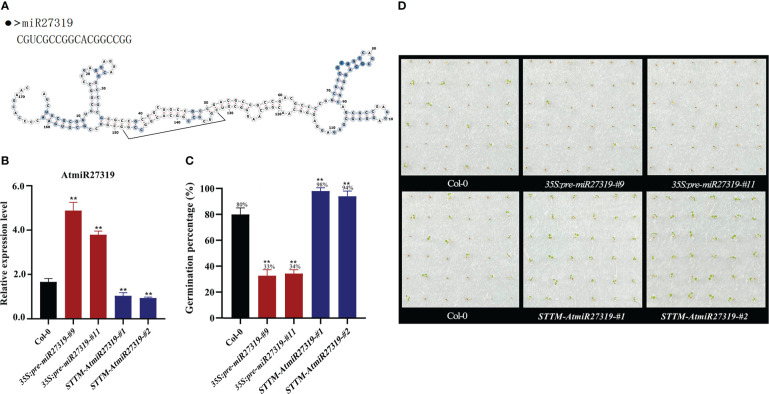
Positive roles of miR27319 in regulating seed dormancy in *Arabidopsis*. **(A)** The sequence and secondary structure of miR27319. **(B)** Relative expression of miR27319 in Col-0, *35S:pre-miR27319*, and *STTM-AtmiR27319*. Significant differences were determined using Student’s t-test: **P < 0.01. **(C)** Seed germination percentages of Col-0, overexpression plants (*35S:pre-miR27319*), and STTM plants of miR27319 (*STTM-AtmiR27319*) in *Arabidopsis*. **(D)** The seed images of Col-0, overexpression plants (*35S:pre-miR27319*), and STTM plants of miR27319 (*STTM-AtmiR27319*) in *Arabidopsis*.

To preliminarily validate the role of miR27319 in SD, miR27319 expression patterns were first verified through qRT-PCR (**Figures 4A**, **S8A**). MiR27319 shared the high similar sequence between wheat and rice, and had three base differences between wheat and *Arabidopsis* (**Figures S9A, B**). Then the miR27319 precursor was heterologously overexpressed in *Arabidopsis* and rice, the mature transcripts of miR27319 in *Arabidopsis* and rice were simultaneously silenced using the STTM method.

Two overexpression lines (*35S:pre-miR27319-#9* and *35S:pre-miR27319-#11*) and two STTM lines of miR27319 (*STTM-AtmiR27319-#1* and *STTM-AtmiR27319-#2*) in *Arabidopsis* were generated. The *pre-miR27319-OE* transcript levels were higher in overexpression lines than in Col-0, and the mature transcripts of miR27319 were lower in the STTM lines than in Col-0 (**Figure 4B**). Seed GPs of miR27319 overexpression lines and STTM lines of *Arabidopsis* were determined. According to the results, the GPs of *35S:pre-miR27319-#9* (average GP 33%) and *35S:pre-miR27319-#11* (average GP 34%) were lower than those of Col-0 (average GP 80%), whereas the GPs of *STTM-AtmiR27319-#1* (average GP 98%) and *STTM-AtmiR27319-#2* (average GP 94%) were higher than those of Col-0 (average GP 80%) (**Figures 4C, D**). This suggests the positive role of miR27319 in SD.

**Figure 5:**
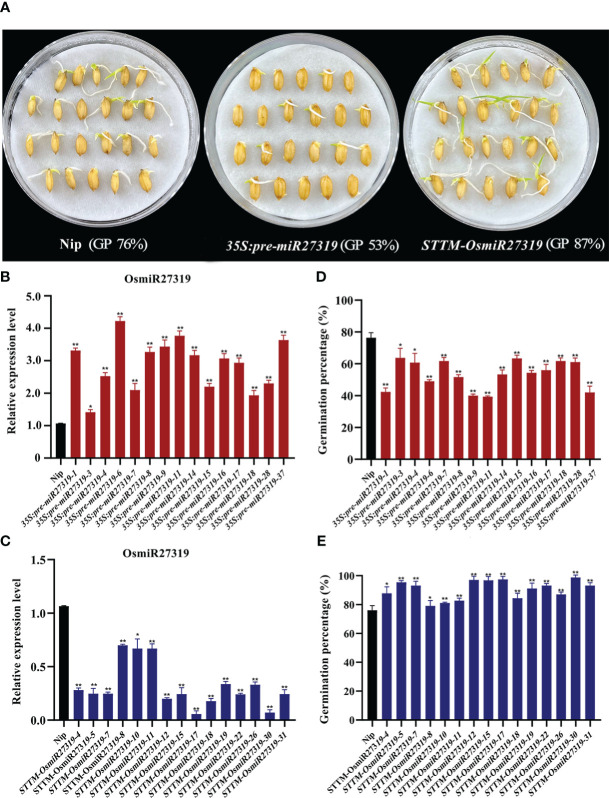
Positive roles of miR27319 in regulating seed dormancy in rice. **(A)** The seed images of Nipponbare (Nip, WT), overexpression plants (*35S:pre-miR27319*), and STTM plants of miR27319 (*STTM-OsmiR27319*) at 7 days after imbibition in rice. **(B)** Relative expression of Nip and *35S:pre-miR27319*. RNA was extracted from seeds imbibed for 24 h. Data are the mean ± standard error (SE). Significant differences were determined using Student’s t-test: **P < 0.01, *P < 0.05. **(C)** Relative expressions of Nip and *STTM-OsmiR27319*. RNA was extracted from seeds imbibed for 24 h. Data are the mean ± standard error (SE). Significant differences were determined using Student’s t-test: ** *P* < 0.01, * *P* < 0.05. **(D)** Germination percentages of Nip and *35S:pre-miR27319*. Data are the mean ± standard error (SE), n = 10–15. Significant differences were determined using Student’ s t-test: ** *P* < 0.01, * *P* < 0.05. **(E)** Germination percentages of Nip and *STTM-OsmiR27319*. Data are the mean ± standard error (SE), n = 10–15. Significant differences were determined using Student’ s t-test: **P < 0.01, *P < 0.05.

Similarly, 15 pre-miR27319 overexpression rice (Nip, WT) lines (*35S:pre-miR27319*) and 15 miR27319 STTM rice lines (*STTM-OsmiR27319*) were generated (**Figures 5A–C**). qRT-PCR analysis revealed that the primary transcripts of miR27319 were higher in the overexpression plants than in the WT lines (**Figure 5B**), and the mature transcripts of miR27319 were lower in the STTM lines than in the WT lines (**Figure 5C**). Compared with the WT lines (average GP 76%), 15 pre-miR27319 overexpression lines exhibited lower GPs (39%–63%, average GP 53%) (*P* < 0.05), whereas 15 STTM-OsmiR27319 lines exhibited higher GPs (80%–98%, average GP 87%) (*P* < 0.05) (**Figures 5D, E**). The linear regression analysis revealed that the relative expression of miR27319 in *35S:pre-miR27319* and *STTM-OsmiR27319* lines had a significant negative correlation with GPs (R^2^ = 0.78** and R^2^ = 0.77**, respectively (**Figures S8B, C**), indicating that miR27319 positively regulates SD in rice.

**Figure 6:**
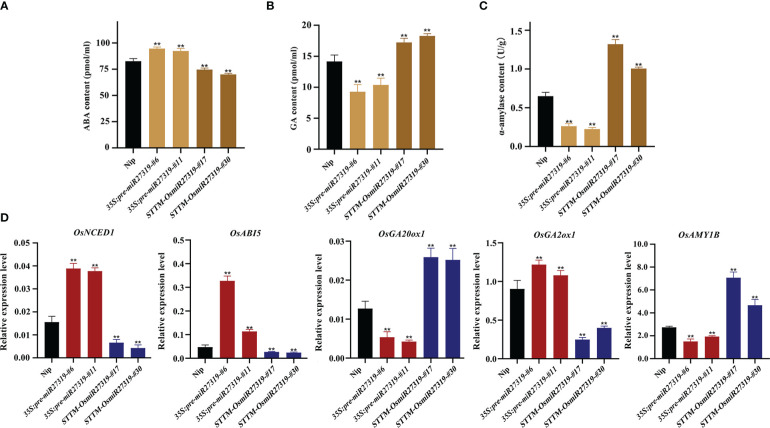
Regulatory pathways of miR27319 in rice seed dormancy. **(A)** ABA contents in Nip, *35S:pre-miR27319*, and *STTM-OsmiR27319*. Significant differences were determined using Student’s t-test: **P < 0.01. **(B)** GA contents in Nip, *35S:pre-miR27319*, and *STTM-OsmiR27319*. Significant differences were determined using Student’s t-test: **P < 0.01. **(C)** Alpha-amylase activities in Nip, *35S:pre-miR27319*, and *STTM-OsmiR27319*. Significant differences were determined using Student’s t-test: **P < 0.01. **(D)** Relative expression levels of the key genes involved in ABA and GA biosynthesis, catabolism, and signaling pathways. Gene ID: *Os03g0645900* (*OsNCED1*), *Os01g0859300* (*OsABI5*), *Os03g0856700* (*OsGA20ox1*), *Os05g0158600* (*OsGA2ox1*), and *Os01g0357400* (*OsAmy1B*). Significant differences were determined using Student’s t-test: **P < 0.01.

Phytohormones GA and ABA are the core factors determining SD and seed germination in diverse plant species. GA promotes seed germination by elevating α-amylase activity, whereas ABA inhibits germination and induces dormancy (Ali-Rachedi et al., 2004; Kucera et al., 2005). To preliminarily explore the molecular mechanism of miR27319 in SD regulation in rice, we first measured the ABA and GA contents as well as α-amylase activity in the seeds of the representative *35S:pre-miR27319-#6*, *35S:pre-miR27319-#11*, *STTM-OsmiR27319-#17*, and *STTM-OsmiR27319-#30* lines as well as in the WT seeds. The seeds of *35S:pre-miR27319-#6* and *35S:pre-miR27319-#11* lines had higher ABA levels and lower GA levels and α-amylase activity than the WT seeds (**Figures 6A–C**). The seeds of *STTM-OsmiR27319-#17* and *STTM-OsmiR27319-#30* lines showed the opposite trends (**Figures 6A–C**). Subsequently, we investigated the expression of several key genes involved in ABA and GA biosynthesis/catabolism/signaling pathways in the seeds of *35S:pre-miR27319* and *STTM-OsmiR27319* as well as the WT plants. Three key genes involved in the ABA biosynthesis/signaling pathways (*OsNCED1* and *OsABI5*) and the GA catabolism pathway (*OsGA2ox1*) exhibited upregulated expression in the seeds of *35S:pre-miR27319* and downregulated expression in the seeds of *STTM-OsmiR27319* compared with the WT seeds (**Figure 6D**). By contrast, the expression of the GA biosynthesis gene *OsGA20ox1* and the α-amylase gene *OsAMY1B* exhibited opposite trends (**Figure 6D**). The results suggest that miR27319 regulates SD through the ABA and GA biosynthesis/catabolism/signaling pathways.

## 4 Discussion

HT during seed maturation has become a serious threat to wheat production because it decreases the grain yield as well as reduces SD, making the plants prone to PHS damage under prolonged rainfall and wet weather before harvest (Lunn et al., 2002; Barnard and Smith, 2009). However, the complicated mechanism of HT-mediated SD remains unclear. High-throughput transcriptome sequencing is a powerful tool for discovering coding and non-coding RNAs involved in HT-mediated SD in the whole genome and is especially useful for studying complex regulatory networks.

Here, using whole-transcriptome sequencing, numerous coding and non-coding RNAs (including 5128 mRNAs, 273 lncRNAs, 21 circRNAs, and 136 miRNAs) were identified to respond to HT in wheat landrace WTB seeds. The common features of lncRNA, miRNA and circRNA are consistent with reported ncRNAs in wheat, confirming the quality of the data (Ma et al., 2015; Sablok et al., 2016; Zhang et al., 2016; ). Then, a predicted regulatory network was constructed on the basis of these differentially expressed coding and non-coding RNAs. This indicates that a complex regulatory mechanism is involved in HT-mediated SD. The above differentially expressed mRNAs were mainly enriched in benzoxazinoid biosynthesis, starch and sucrose metabolism, and protein processing in the endoplasmic reticulum and in amino sugar, consistent with previous transcriptome data of dormancy (Kato-Noguchi, 2008; Liew et al., 2020; Zhang et al., 2021). Interestingly, some pathways found in the present study, including nucleotide sugar metabolism and valine, leucine, and isoleucine biosynthesis, have not yet been reported to be associated with SD; these findings need to be validated in the future. Of note, abundant transcripts of the identified novel miRNAs, lncRNAs, and circRNAs will allow deep dissection of the complicated regulatory network of HT-mediated SD in wheat.

According to previous studies, *MFT* and *DOG1* play important roles in SD in wheat and *Arabidopsis*, respectively, and the expression of these two genes are upregulated at a low temperature, resulting in increased dormancy levels (Nakamura et al., 2011; Graeber et al., 2014). In the present study, the expression of *TaMFT* and *TaDOG1* and their homologs (*TaMFT-3A*, *TaMFT-3D*, *TaDOG1-3A*, and *TaDOG1-3D*) was validated to be down regulated by HT during seed maturation. This suggests that HT mediates SD partially by influencing the expression of the known dormancy genes.

In addition, several key genes involved in the ABA and GA biosynthesis/signaling pathways (*TaSnRK2-2D*, *TaAFP-2D*, *GID1-1A*, *GID1-1D*, and *TaGA20ox1*) were found to respond to HT, demonstrating the vital roles of ABA and GA in HT-mediated dormancy. To further verify the findings, we determined the GPs of the seeds of the EMS mutant *ga20ox1* of *TaGA20ox1* in the wheat cultivar JM22 background under NT and HT environments. The GPs of the *ga20ox1* mutant seeds was significantly reduced under the HT environment compared with those of the WT seeds, indicating that the *ga20ox1* mutant is less sensitive to HT. These findings support that *TaGA20ox1* is involved in HT-mediated SD in wheat. Interestingly, *TaGA20ox1* mutation in the *ga20ox1* mutant did not adversely affect other yield-related traits, such as grain weight and size, although plant height decreased slightly (**Figure S6B**). Moreover, to evaluate the application potentiality in wheat breeding to improve PHS resistance, we investigated the germination behaviors of the *ga20ox1* mutant and JM22 seeds in the 3rd week of afterripening. Their GP values reached 100% on the 3rd day of the germination test, implying that the *ga20ox1* mutation does not affect germination speed and the uniformity of seedling emergence and subsequent maturity (**Figure S6C**). Thus, *TaGA20ox1* may be a valuable target for breeding wheat varieties with high PHS resistance in a HT environment.

Calcium is a critical secondary messenger involved in ABA signal transduction (Himmelbach et al., 2003). Ca^2+^-dependent protein kinases (CDPKs or CPKs), being one of the calcium sensory proteins, are important components in ABA signaling (Ludwig et al., 2004). Several *CDPK* genes have been proven to be involved in seed germination. For example, *Arabidopsis CPK32* was shown to positively regulate ABA-regulated seed germination (Choi et al., 2005); *Arabidopsis CPK4* and *CPK11*, two homologous *CDPK*s, positively regulated ABA-mediated seed germination (Zhu et al., 2007); and *Arabidopsis CPK12* negatively regulated ABA signaling-mediated seed germination and post-germination growth (Zhao et al., 2011). These findings indicate that different *CDPK* family members regulate SD and seed germination by positively or negatively regulating ABA signal transduction. In the present study, the expression of *TaCDPK21*, one of the *CDPK* family members, was upregulated by HT, implying its negative role in SD and positive role in germination. To verify the findings, we assessed the GP of the loss-of-function EMS mutant of *TaCDPK21* in the wheat cultivar JM22 background. The GP significantly reduced and dormancy obviously increased, indicating the negative role of *TaCDPK21* in SD. Moreover, *TaCDPK21* overexpression *Arabidopsis* and functional complementarity tests supported the afore mentioned results. Intriguingly, *TaCDPK21* silencing had no obvious adverse effects on other vital agronomic traits, such as plant height, grain weight, grain size, and spike shape (data not shown). To evaluate the application potentiality in wheat breeding for improving PHS resistance, we investigated the germination behaviors of the *cdpk21* mutant and JM22 seeds in the 3rd week of afterripening. Their GP values reached 99%–100% on the 3rd day of the germination test, implying that the *cdpk21* mutant does not affect germination speed and the uniformity of seedling emergence and subsequent maturity (**Figure S7D**). Therefore, we speculate that *TaCDPK21* is a potential target for developing wheat varieties with high dormancy and PHS resistance that can cope with prolonged rainfall and wet weather during the harvest season.

By targeting mRNAs for cleavage or inhibiting their translation, miRNAs act as important regulators in plant development and stress responses by negatively modulating gene expression at the post-transcriptional level (Zhang et al., 2015). Studies have proven that many miRNAs are associated with SD and seed germination in different plant species (Liu et al., 2007; Kim et al., 2010; Huo et al., 2016; Guo et al., 2018; Miao et al., 2019; Jiang et al., 2021). In particular, Jiang et al. (2021) reported that *PHYTOCHROME INTERACTING FACTOR 1* (*PIF1*) repressed miR408 accumulation by binding to the miR408 promoter, thereby post-transcriptionally modulating *PLANTACYANIN* (*PCY*) abundance for controlling light-dependent seed germination in *Arabidopsis*. This shows that miR408 plays a positive role in seed germination. We here also identified numerous miRNAs related to HT-regulated SD in WTB seeds. Among them, miR408 expression was downregulated by HT, implying its positive role in SD and a negative role in germination, which is not in accordance with the results of Jiang et al. (2021). Moreover, the target genes of miR408 reported by Jiang et al. (2021) were not identified in the present study. This finding suggests that diverse regulatory networks of miR408 may be involved in light- and temperature-mediated germination in different plant species. Intriguingly, in the current study, *TaCDPK21*, which was negatively associated with dormancy, was predicted as one of the miR408 targets. However, more experiments are required to confirm the cleavage relationship between miR408 and *TaCDPK21*, as well as their functions in HT-mediated SD in wheat.

In addition, a novel miR27319, located at the key node of the regulatory network, was confirmed to be downregulated by HT in this study. Using miR27319 overexpression and STTM lines of *Arabidopsis* and rice, we validated the positive role of miR27319 in SD, suggesting that the functions of miR27319 are conserved between monocots and dicots. Presently, we are conducting the functional validation experiments of miR27319 in common wheat. The miRNAs and their targets coordinate together to form diverse miRNA–target modules to perform their different biological functions in plants (Zhang, 2015; Xu et al., 2021). In the present study, miR27319 was predicted to have multiple targets, mainly including three peroxidase genes (*TaPer46-2A*, *TaPer64-2A*, and *TaPer64-2D*) (**Figure S8D**). Peroxiredoxins (Prxs) are central elements of the antioxidant defense system that can eliminate excessive reactive oxygen species (ROS) and thus alleviate oxidative damage. According to the cysteine number and position, Prxs can be categorized into four classes: 1-Cys Prx, typical 2-Cys Prx, atypical 2-Cys Prx (type-II Prx), and Prx Q (Dietz, 2011). Many studies have shown that Prxs (especially 1-Cys Prx) have crucial roles in regulating SD and seed germination in different plants, such as *pBS128* (1-Cys Prx, also named *PER1*) in bromegrass (Goldmark et al., 1992), *Per1* (homologous to a dormancy-related transcript *pBS128*) in barley (Stacy et al., 1996), *AtPer1* in *Arabidopsis* (Haslekas et al., 1998; Chen H et al., 2020), and *NnPER1* in *Nelumbo nucifera* (Chen et al., 2016). We speculate that *TaPer46* and *TaPer64* participate in HT-mediated SD through the ROS pathway, and their functions in wheat and their interaction with miR27319 need to be validated in the future.

In summary, we identified numerous known and novel coding and non-coding RNAs involved in HT-regulated SD during development in wheat landrace WTB through whole-transcriptome sequencing. We also validated the expression patterns of some of these RNAs through qRT-PCR. Combining the results of transgenic experiments in *Arabidopsis* and rice and EMS mutant analysis in wheat, we confirmed the roles of *TaGA20ox1*, *TaCDPK21*, and miR27319 in SD. Our data broadened our understanding of the complex regulatory network of HT-mediated dormancy and provide new gene resources for improving PHS resistance to minimize PHS damage in wheat.

## Data availability statement

The data were deposited in the National Center for Biotechnology Information Databank (NCBI) (accession number: PRJNA895954) (https://www.ncbi.nlm.nih.gov/bioproject/PRJNA895954/).

## Author contributions

HJ, WG, CC, and HZ conceived the project. JL and C-XM provided materials. CM, CC and HZ acquired funding. HJ, WG, BJ, XL and Y-TJ designed the plot layout and planned the study. HJ, WG, L-TZ, YZ, SY, J-JC and LJ performed field activities. HJ, WG, B-LJ and XL analyzed the data. HJ wrote a draft of the manuscript. WG, CC and H-PZ supervised the study. All authors contributed to the article and approved the submitted version.
