# Supplementary material for: Identification and validation of coding and non-coding RNAs involved in high-temperature-mediated seed dormancy in common wheat
Source: Front Plant Sci. 2023 Feb 1;14:1107277. doi: 10.3389/fpls.2023.1107277 (PMC9929302; doi:10.3389/fpls.2023.1107277)
Supplement: Supplementary file 1 [file DataSheet_1.docx]

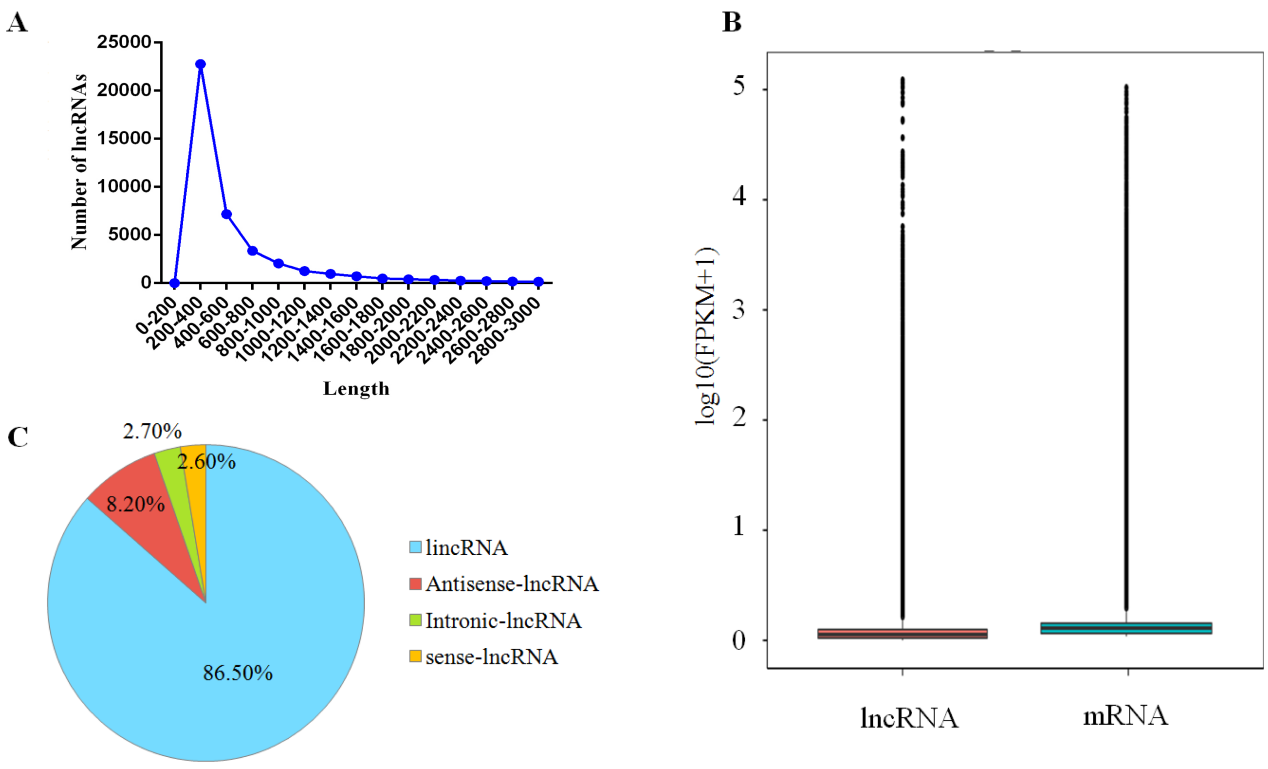


**Fig. S1** Length distribution, expression, and classes of lncRNAs and mRNAs

**A** Length distribution of lncRNAs. **B** Box plots of FPKM for lncRNAs and mRNAs involved in all samples. **C** Distribution graphs for different types of lncRNAs.


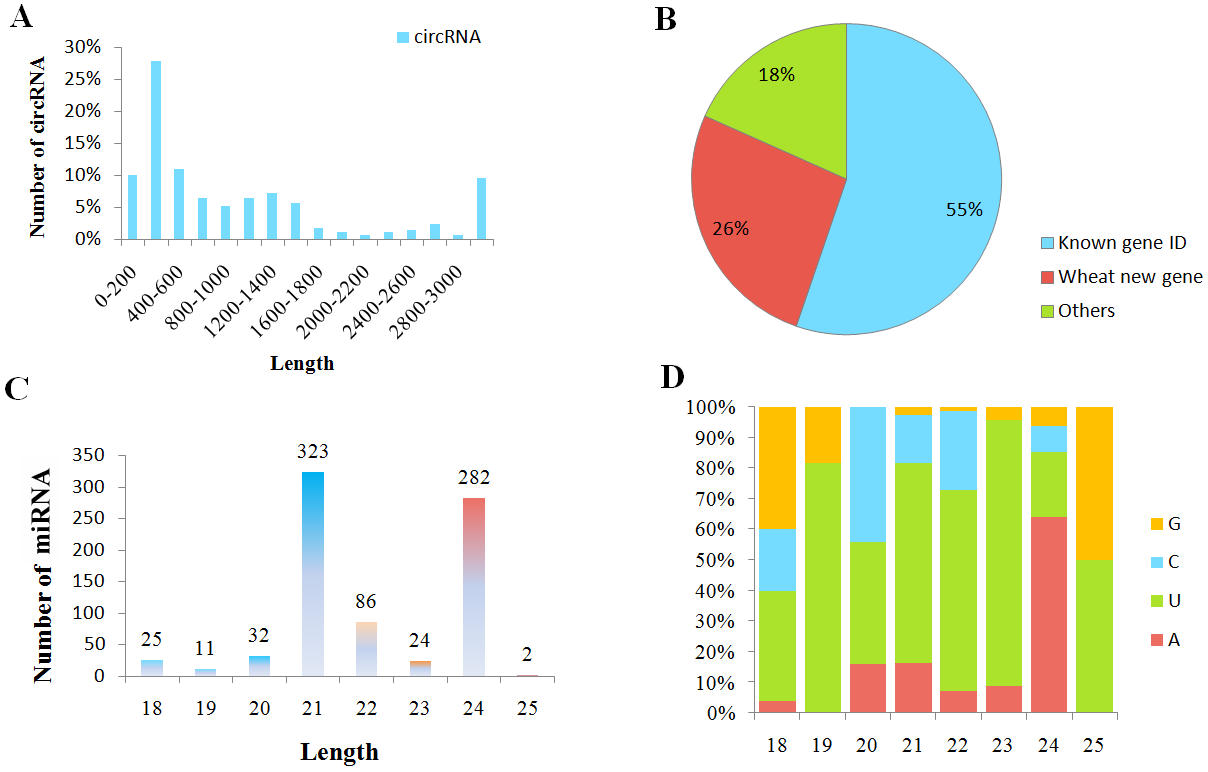


**Fig. S2** Descriptions of circRNAs and miRNAs

**A** Length distribution of circRNAs. **B** Distribution graphs for different types of circRNAs based on derived transcripts. **C** Length distribution of miRNAs. **D** The proportions of the first base distribution of miRNAs.


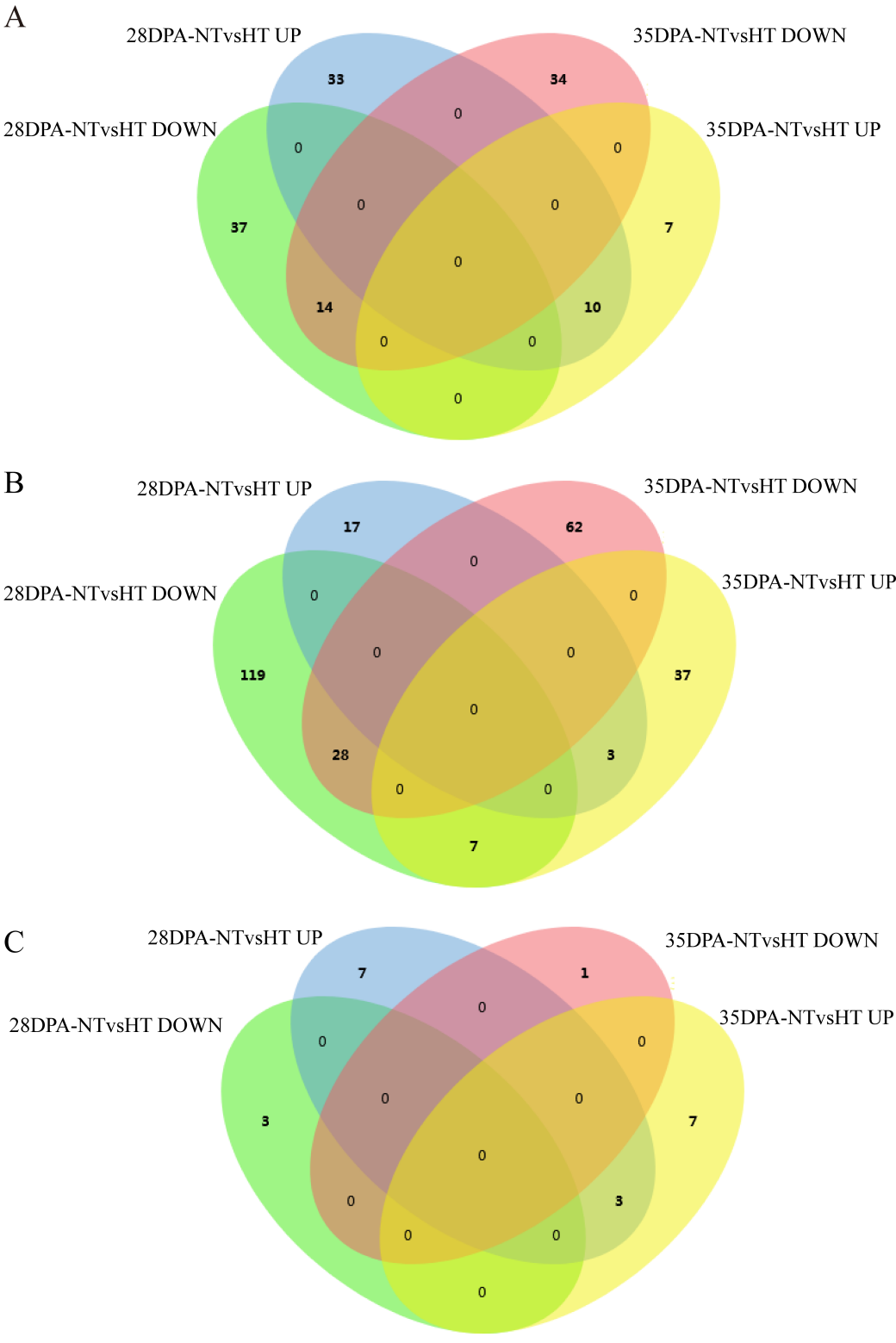


**Fig. S3** Differentially expressed miRNAs, lncRNAs, and circRNAs

**A** Venn diagram of differentially expressed miRNAs. **B** Venn diagram of differentially expressed lncRNAs. **C** Venn diagram of differentially expressed circRNAs.


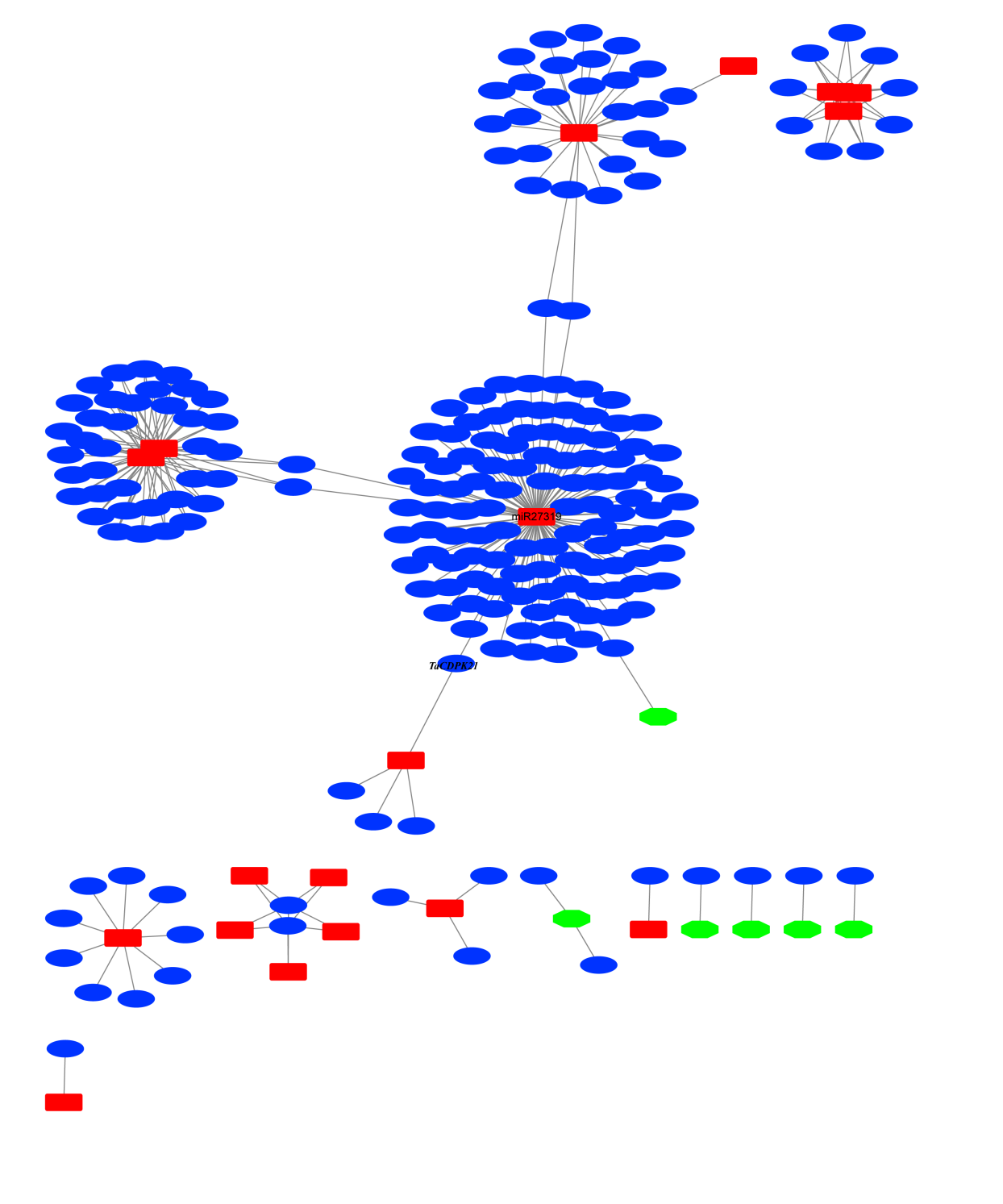


**Fig. S4** [Regulatory](file:///C:\Users\jiangh\AppData\Local\youdao\dict\Application\7.5.2.0\resultui\dict\?keyword=regulatory) [network](file:///C:\Users\jiangh\AppData\Local\youdao\dict\Application\7.5.2.0\resultui\dict\?keyword=network) of mRNAs and ncRNAs involved in high temperature-mediated seed dormancy

The red squares represent miRNAs, the blue ellipses represent mRNAs, and the green hexagons represent lncRNAs.


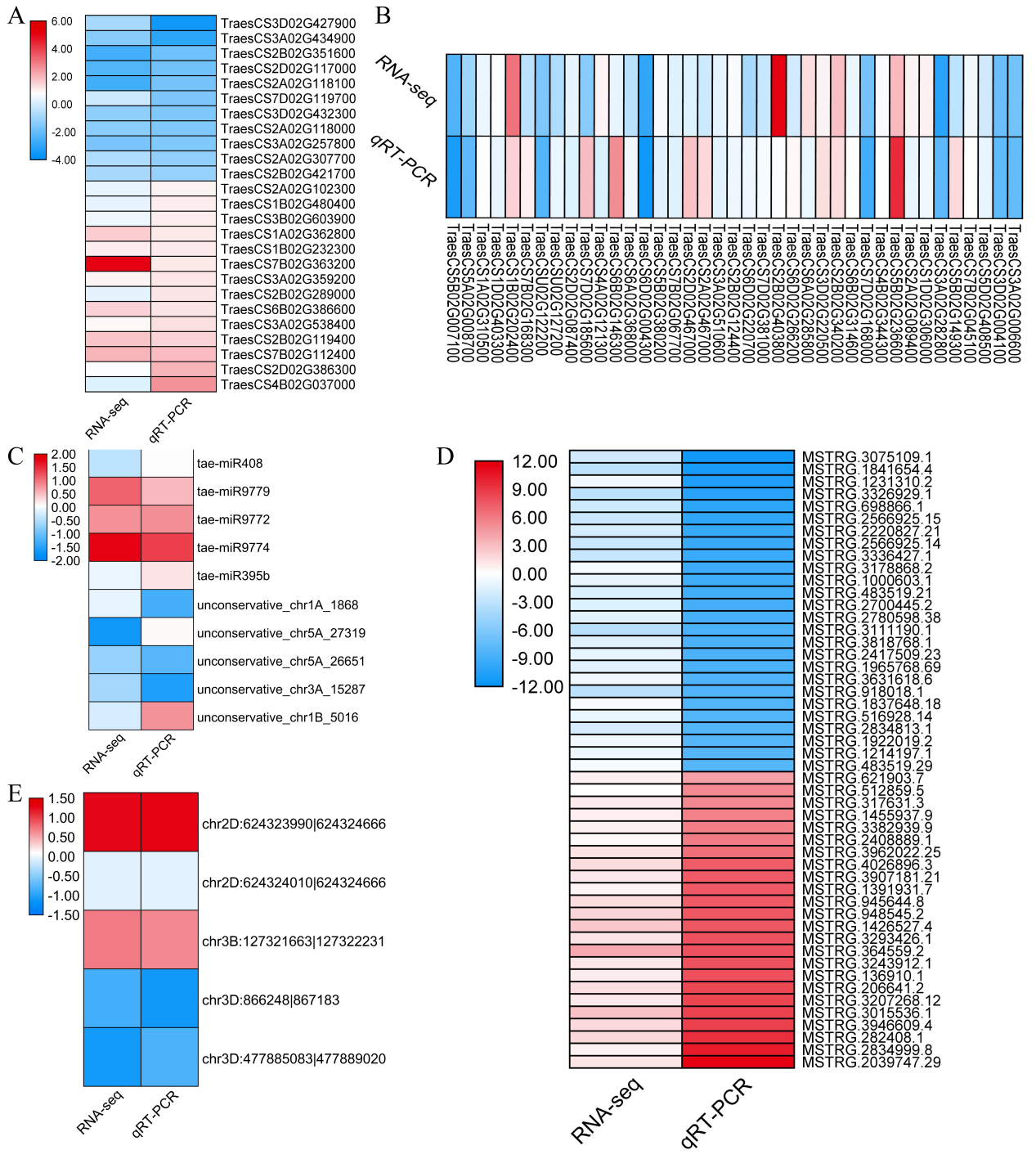


**Fig. S5** Expression patterns of the selected mRNAs, miRNAs, lncRNAs, and circRNAs in 35DPA-NTvsHT determined through RNA-seq and qRT-PCR

**A** Expression patterns of the selected transcription factors determined through RNA-seq and qRT-PCR approaches. **B** Expression patterns of the selected seed dormancy-related mRNAs determined through RNA-seq and qRT-PCR. **C** Expression patterns of the selected miRNAs determined through RNA-seq and qRT-PCR. **D** Expression patterns of the selected lncRNAs determined through RNA-seq and qRT-PCR. **E** Expression patterns of the selected circRNAs determined through RNA-seq and qRT-PCR.


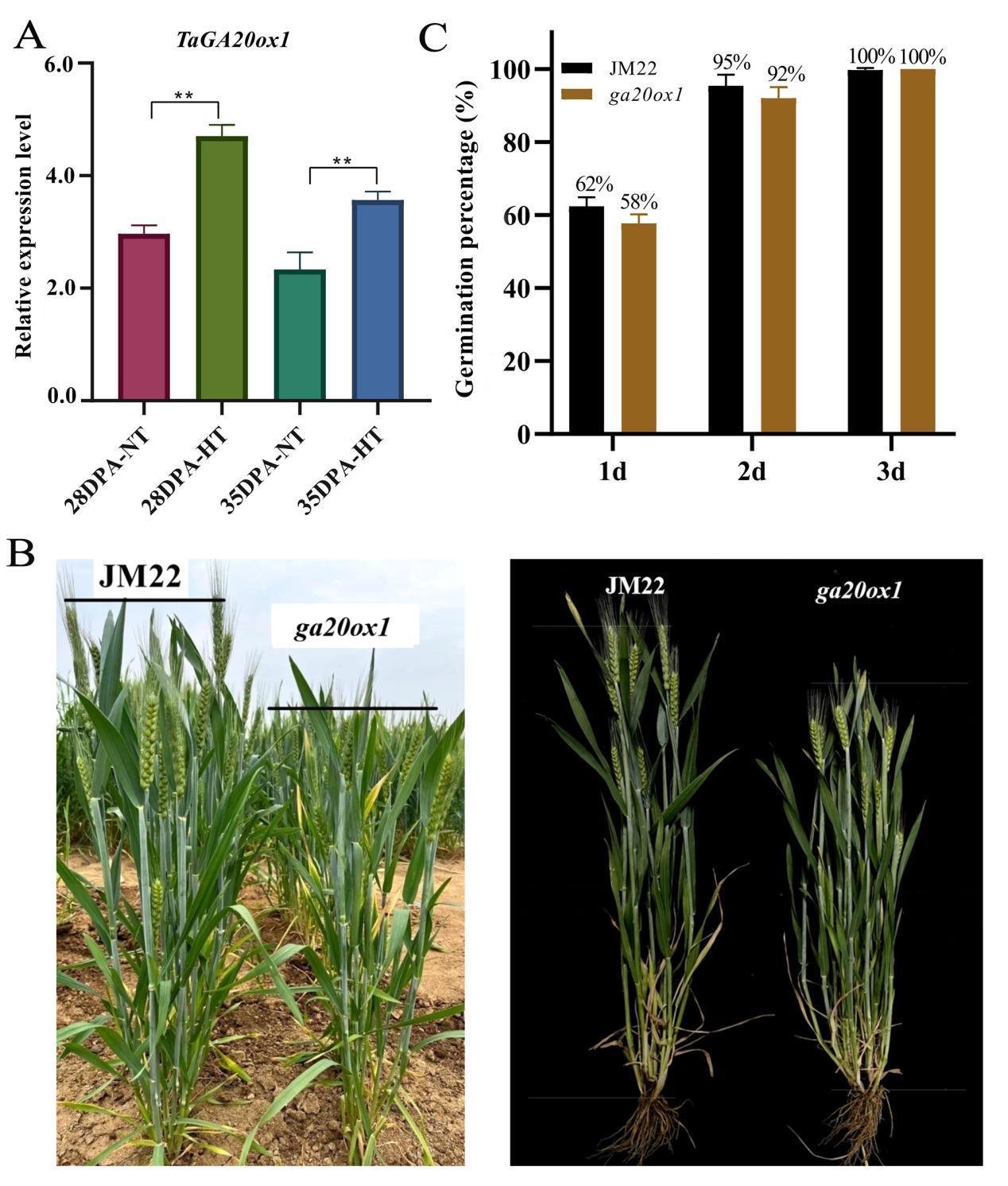


**Fig. S6** Relative expression, plant images and germination information of *TaGA20ox1*

**A** Relative expression of *TaGA20ox1* determined through qRT-PCR. Significant differences were determined using Student’s t-test: ** *P* < 0.01.**B** The plant images of Jimai 22 (JM22) and *ga20ox1* during development. **C** Germination percentages of Jimai 22 (JM22) and the EMS mutant *ga20ox1* in the 3rd week of afterripening conducted at normal temperature (25°C/20°C). Data represent the mean ± standard error (SE), n = 10–15.


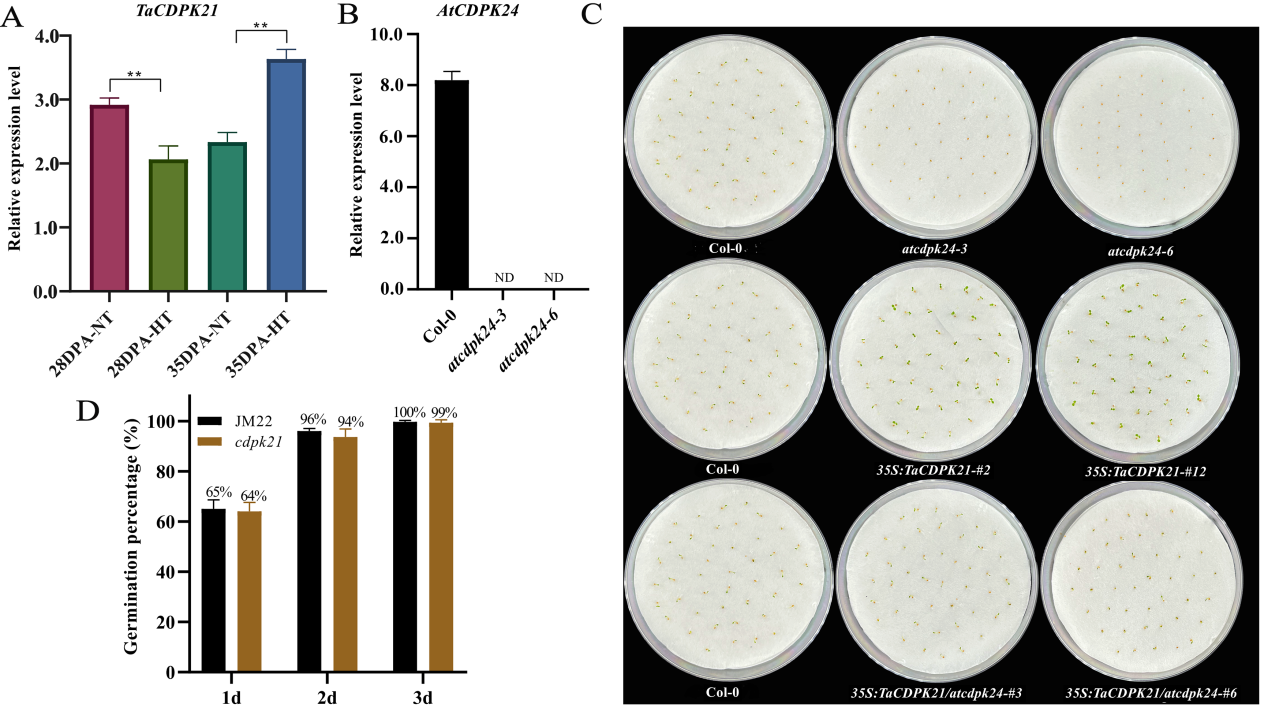


**Fig. S7** Relative expression, seed images and germination information of *TaCDPK21*

**A** Relative expression of *TaCDPK21* determined through qRT-PCR. Significant differences were determined using Student’s t-test: ** *P* < 0.01. **B** Relative expression of *AtCDPK24* in Col-0 and the T-DNA mutant *atcdpk24*. RNA was extracted from seeds imbibed for 24 h. ND represents not detected. **C** The seed images in Col-0, overexpression *Arabidopsis* plants (*35S:TaCDPK21*), and complementation of *TaCDPK21* (*35S:TaCDPK21/atcdpk24*). **D** Germination percentages of Jimai 22 (JM22) and the EMS mutant *cdpk21* in the 3rd week of afterripening conducted at normal temperature (25°C/20°C). Data represent the mean ± standard error (SE), n = 10–15.


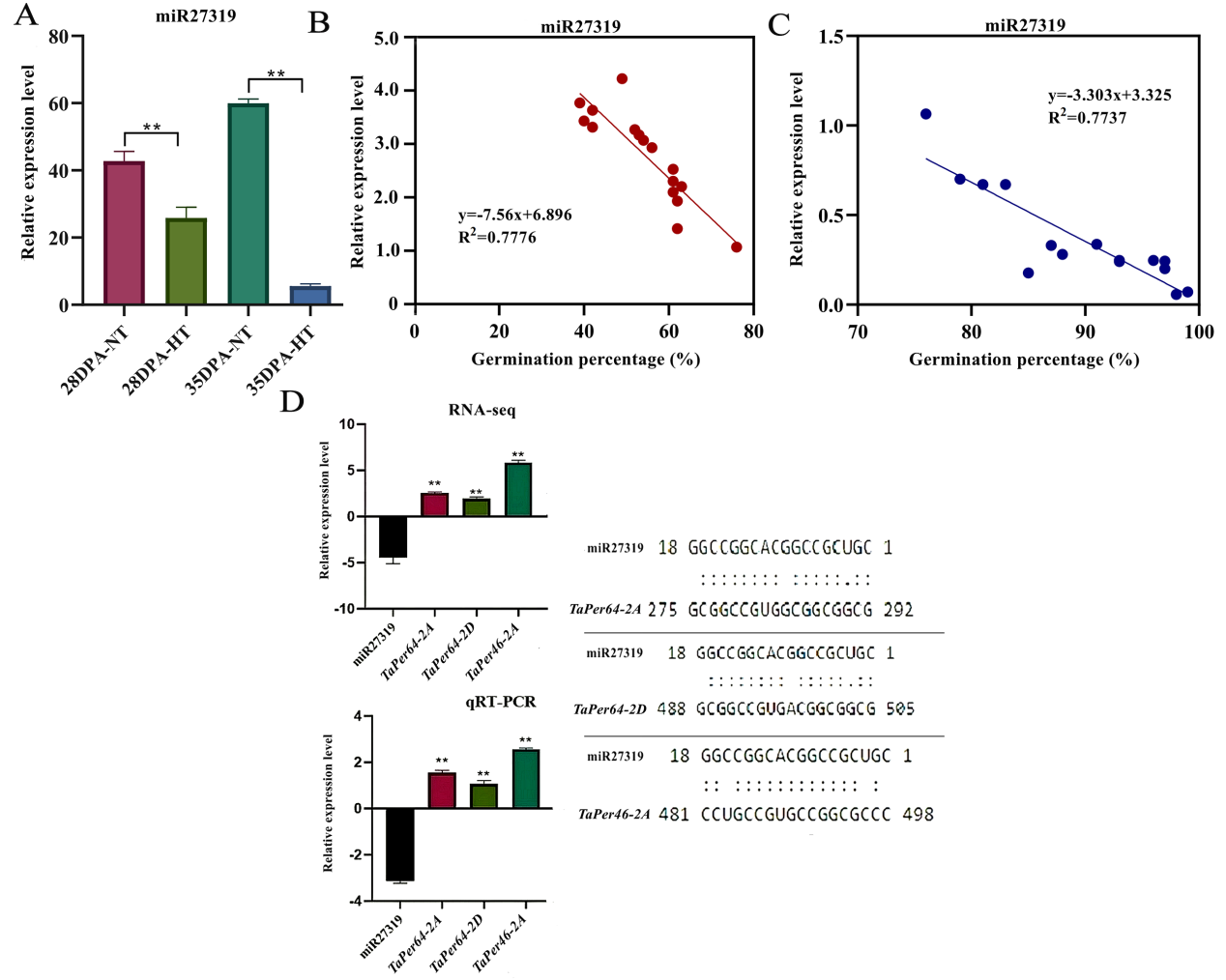


**Fig. S8** Relative expressions and target genes of miR27319

**A** Relative expression of miR27319 determined through qRT-RCR. **B** Correlation coefficient between the expression level and seed germination of Nip and *35S:pre-miR27319*. **C** Correlation coefficient between the expression level and seed germination of Nip and *STTM-OsmiR27319*. **D** Expression and sequence pairing of miR27319 and its target genes. Gene ID: *TraesCS2A02G467000* (*TaPer64-2A*), *TraesCS2D02G467000* (*TaPer64-2D*), and *TraesCS2A02G355300* (*TaPer46-2A*).


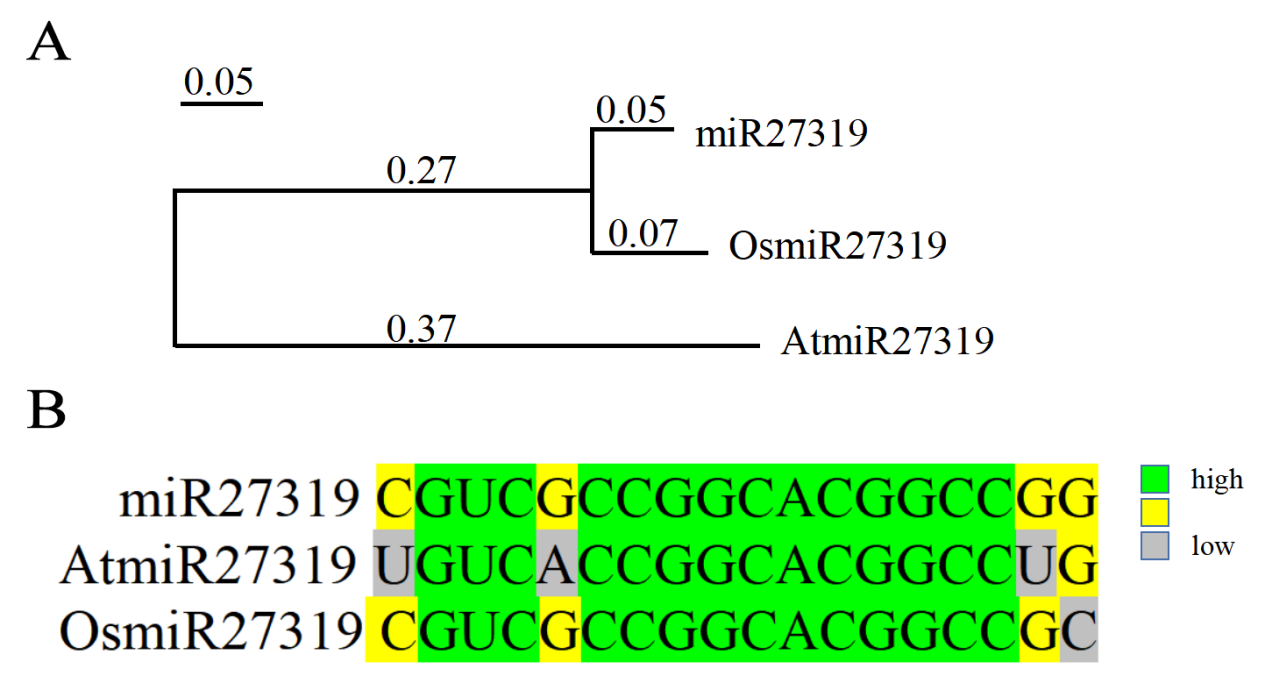
**Fig. S9** Phylogenic tree analysis and sequence of miR27319 in wheat, rice, and *Arabidopsis*

**A** Phylogenic tree analysis of miR27319 in wheat, rice, and *Arabidopsis.* **B** Mature sequence of miR27319 in wheat, rice, and *Arabidopsis.*
